# CT Reconstruction Kernels and the Effect of Pre- and Post-Processing on the Reproducibility of Handcrafted Radiomic Features

**DOI:** 10.3390/jpm12040553

**Published:** 2022-03-31

**Authors:** Turkey Refaee, Zohaib Salahuddin, Yousif Widaatalla, Sergey Primakov, Henry C. Woodruff, Roland Hustinx, Felix M. Mottaghy, Abdalla Ibrahim, Philippe Lambin

**Affiliations:** 1 The D-Lab, Department of Precision Medicine, GROW-School for Oncology, Maastricht University, 6200 Maastricht, The Netherlands; z.salahuddin@maastrichtuniversity.nl (Z.S.); y.widaatalla@maastrichtuniversity.nl (Y.W.); s.primakov@maastrichtuniversity.nl (S.P.); h.woodruff@maastrichtuniversity.nl (H.C.W.); a.ibrahim@maastrichtuniversity.nl (A.I.); philippe.lambin@maastrichtuniversity.nl (P.L.); 2 Department of Diagnostic Radiology, Faculty of Applied Medical Sciences, Jazan University, Jazan 45142, Saudi Arabia; 3 Department of Radiology and Nuclear Medicine, Maastricht University Medical Center, 6200 Maastricht, The Netherlands; f.mottaghy@mumc.nl; 4 Division of Nuclear Medicine and Oncological Imaging, Department of Medical Physics, University Hospital of Liege and GIGA CRC-In Vivo Imaging, University of Liege, 4000 Liege, Belgium; rhustinx@chu.ulg.ac.be; 5 Department of Nuclear Medicine and Comprehensive Diagnostic Center Aachen (CDCA), University Hospital RWTH Aachen University, 52074 Aachen, Germany; 6 Department of Radiology, Columbia University Irving Medical Center, New York, NY 10032, USA

**Keywords:** radiomics reproducibility, reconstruction kernel, ComBat harmonization, image harmonization

## Abstract

Handcrafted radiomics features (HRFs) are quantitative features extracted from medical images to decode biological information to improve clinical decision making. Despite the potential of the field, limitations have been identified. The most important identified limitation, currently, is the sensitivity of HRF to variations in image acquisition and reconstruction parameters. In this study, we investigated the use of Reconstruction Kernel Normalization (RKN) and ComBat harmonization to improve the reproducibility of HRFs across scans acquired with different reconstruction kernels. A set of phantom scans (*n* = 28) acquired on five different scanner models was analyzed. HRFs were extracted from the original scans, and scans were harmonized using the RKN method. ComBat harmonization was applied on both sets of HRFs. The reproducibility of HRFs was assessed using the concordance correlation coefficient. The difference in the number of reproducible HRFs in each scenario was assessed using McNemar’s test. The majority of HRFs were found to be sensitive to variations in the reconstruction kernels, and only six HRFs were found to be robust with respect to variations in reconstruction kernels. The use of RKN resulted in a significant increment in the number of reproducible HRFs in 19 out of the 67 investigated scenarios (28.4%), while the ComBat technique resulted in a significant increment in 36 (53.7%) scenarios. The combination of methods resulted in a significant increment in 53 (79.1%) scenarios compared to the HRFs extracted from original images. Since the benefit of applying the harmonization methods depended on the data being harmonized, reproducibility analysis is recommended before performing radiomics analysis. For future radiomics studies incorporating images acquired with similar image acquisition and reconstruction parameters, except for the reconstruction kernels, we recommend the systematic use of the pre- and post-processing approaches (respectively, RKN and ComBat).

## 1. Introduction

Recent decades have witnessed an exponentially increasing number of studies investigating the potential of quantitative imaging features to extract additional information from medical images not detectable by human eyes [1,2]. Handcrafted radiomics refers to the high-throughput extraction of quantitative imaging features from medical images to decode biologic information [3,4] and, today, more than 5000 studies can be returned on the PubMed database using “radiomics” as a search word. The handcrafted radiomics approach “involves manual segmentation of the region of interest (e.g., the tumor) on medical imaging and extraction of thousands of human-defined and curated quantitative features from the region of interest” [5].

The hypothesis in radiomics studies is that handcrafted radiomic features (HRFs) can be used singularly or collectively as clinical biomarkers [3]. Many studies have investigated and reported on the potential of HRFs to predict clinical endpoints, such as overall survival [6,7,8], tissue histology [9,10,11,12,13] and response to therapy [14,15]. These studies highlighted the potential of such approaches to be applied in clinical settings, since they could present non-invasive, reliable, readily available and cost-effective alternatives to current invasive clinical procedures, such as tissue biopsies. Moreover, with proper application, radiomics could provide reproducible predictions, which are quantitative and less dependent on the subjective interpretation of medical examinations [16,17].

With the development of handcrafted radiomics as a research field, the limitations the field faces have been increasingly investigated during recent years [4,18]. The most important identified limitation currently is the sensitivity of HRFs to variations in image acquisition and reconstruction parameters [19,20,21,22,23,24]. For an HRF to be used as a clinical biomarker (solely or in combination with other HRFs), it has to be reproducible across different imaging parameters for generalization purposes [24]. However, many studies have reported on the sensitivity of HRFs to variations in time (test–retest) [25,26,27,28,29] and to variations in imaging acquisition and reconstruction parameters [30,31,32,33,34,35,36,37]. Studies have also reported that the degree of variation in a single acquisition or reconstruction parameter affects the reproducibility of HRFs variably [31,34]. A number of studies have reported the significant effects of variations in reconstruction kernels on the reproducibility of HRFs [20,38].

Different methods have been investigated to address the issue of reproducibility of HRFs across scans acquired differently. ComBat harmonization [39] is one of the post-processing methods that have recently been extensively investigated in radiomics analyses [40,41,42]. ComBat harmonization is a method that was developed for removing batch effects—attributed to the use of different machinery—from gene expression arrays. A number of studies have reported on the applicability of ComBat harmonization in different scenarios, such as scans acquired with varying degrees of differences in CT image acquisition and reconstruction parameters, scans acquired with a single variation in an image reconstruction parameter (in-plane resolution) and scans of different contrast-enhancement phases [31,35,43,44]. These studies reported that the performance of ComBat in radiomics analyses is dependent on the variations in the data being harmonized. A number of studies have also investigated the potential of ComBat in different scenarios [45,46,47,48]. However, the potential of ComBat to remove batch effects attributed solely to the variations in the reconstruction kernel has yet to be thoroughly investigated. Other investigated methods include pre-processing of the images to minimize effects due to differences in slice thickness, reconstruction with convolutional kernels, etc. Normalization of chest CT data minimized the variability that resulted from different reconstruction kernels [49]. The authors developed a method that targeted reducing the variations in the quantification of emphysema by normalizing the reconstruction kernel (Reconstruction Kernel Normalization—RKN). The CT scans obtained from different scanners that were reconstructed with varying kernels showed reduced variability in emphysema quantification after the proposed iterative normalization. However, the effect of this normalization method on the reproducibility of HRFs has not been investigated.

In this study, we hypothesize that the use of RKN and ComBat could improve the reproducibility of HRFs across scans acquired with different reconstruction kernels depending on the variations in the data being analyzed and/or harmonized. We further hypothesize that the combination of both methods (RKN and ComBat) would give superior results in terms of “number of reproducible HRFs” compared to no or only one harmonization method. Given that variations in the convolution kernel impact the reproducibility of HRFs the most, we investigate the reproducibility of HRFs extracted from phantom CT scans acquired with different reconstruction kernels on different imaging vendors. We also investigate the potential of ComBat harmonization, RKN and the combination of both methods to reduce the variations in HRF values attributed to differences solely in the reconstruction kernels of the original scans.

## 2. Materials and Methods

### 2.1. Imaging Data

The phantom data used in the study were obtained from the public Credence Cartridge Radiomics (CCR) phantom dataset [50] from the Cancer Imaging Archive site (TCIA.org) [51]. A total of 251 scans were acquired using different scanners, acquisition and reconstruction parameters. For this study, we included scans that were acquired using the same imaging acquisition and reconstruction parameters, except for the convolution kernel. After applying the inclusion criteria, 28 scans from five different scanner models were used in this study (Table 1).

### 2.2. Volume of Interest and HRFs Extraction

Each layer of the phantom was segmented as a single volume of interest (VOI), with the dimensions 8 × 8 × 2 cm^3^. A total of 10 VOIs were segmented per scan, resulting in a total of 280 VOIs. HRFs were extracted using the open source PyRadiomics software version 2.2.0 [52]. HRFs were extracted at two different stages: directly from the original scans; and after image pre-processing. Image intensities were binned in all of the three scenarios with a binwidth of 25 Hounsfield units (HUs) to reduce noise levels and texture matrix sizes and the amount of computational power needed. No other image pre-processing was applied in any of the scenarios. Extracted HRFs were HU intensity features and texture features of five matrices: gray-level co-occurrence (GLCM); gray-level run-length (GLRLM); gray-level size zone (GLSZM); gray-level dependence (GLDM); and neighborhood gray-tone difference (NGTDM) matrices. A more detailed description of PyRadiomics HRFs can be found online at: https://pyradiomics.readthedocs.io/en/latest/features.html (accessed on 13 October 2021).

### 2.3. Reconstruction Kernel Normalization

The CT scan $I_{o}$ is decomposed into a series of frequency components $F^{i}$. Image $I_{o}$ is convoluted with the Gaussian filter at $\sigma_{i}$ scale ($\sigma_{i}$ = 0, 1, 2, 4, 8, 16) to get a filtered image $L_{\sigma_{i}}$. The frequency component for *i* = 0, 1, 2, 3, 4 is given by $F^{i+1}$ = $L_{\sigma +1}\;$− $L_{\sigma_{i+1}}$ and for *i* = 5 it is given by $F^{i+1}$ = $L_{\sigma_{i}}$. The normalized image $I_{N}$ is obtained by $I_{N}$ = $F^{6}$ + $\overset{5}{\underset{i=1}{\sum }}\lambda_{i}\cdot F^{5}$. $\lambda_{i}$ is given by $\frac{r_{i}}{e_{i}}$, where $r_{i}$ and $e_{i}$ are the standard deviations of the intensity values in the band $F^{i}$ of the reference image and image $I_{o}$, respectively. This process is repeated until $\lambda_{i}$ is within the range [0.95, 1.05]. This method was proposed for reducing the effects of varying reconstruction kernels for emphysema quantification in chest CT scans [49]. We investigated the effect of applying this normalization method on feature reproducibility.

### 2.4. Image Pre-Processing and HRF Post-Processing

**Figure 1:**
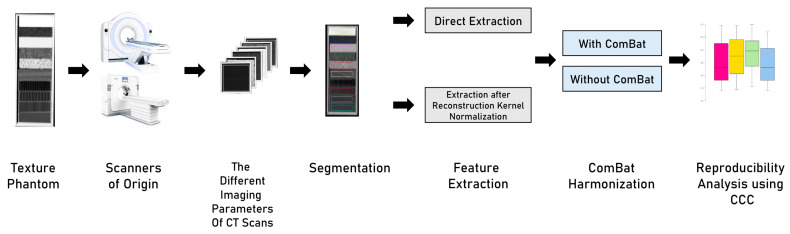
The study workflow.

Four scenarios were analyzed in this study (Figure 1): (i) HRFs extracted from original images; (ii) HRFs extracted from pre-processed scans with the method described in Section 2.3; (iii) HRFs extracted from original images and harmonized with ComBat; and (iv) the combination of both methods. In scenario (ii), image pre-processing was performed using the method previously described in [49]. Each set of images (*n* = 5) was normalized to a reference scan from the set. HRFs were extracted following image pre-processing. In scenario (iii), ComBat harmonization was applied on HRFs extracted from the original scans without pre-processing. ComBat harmonization in radiomics has been previously described [43]. In scenario (iv), HRFs were extracted from images normalized with the RKN method and harmonized using ComBat harmonization.

### 2.5. Statistical Analysis

All statistical analyses were performed using R [53] on RStudio (V3.6.3) [54]. For each scanner model, scans were compared in a pair-wise manner. The concordance correlation coefficient (CCC) was used to assess the reproducibility of HRFs across different pairs [55] (epiR package V. 2.0.26) [56]. The CCC assesses the agreement in the value and rank for each HRF across the pairwise scenarios. HRFs with CCC > 0.9 were considered reproducible in a given scenario. The CCC was calculated in each of the investigated scenarios described in Section 2.4.

To assess the statistical significance of the differences in the number of reproducible HRFs in each scenario, the McNemar test was used [57]. The McNemar test is used to assess whether marginal frequencies are equal before and after an intervention. In this study, we calculated McNemar’s *p*-values using the HRFs extracted from the original images and after RKN, ComBat, and the combination of both. We also calculated the *p*-values among the methods, as well as the *p*-values for each method compared to the combination of methods. For each pair, the difference in the number of reproducible HRFs was labeled “significant” or “not significant” depending on the *p*-value.

## 3. Results

### 3.1. The Effect of Differences in Convolution Kernels on the Reproducibility of HRFs

The Pyradiomics toolbox provides a set of 91 original HRFs from each VOI. These HRFs are divided into First Order Statistics (*n* = 18), GLCM (*n* = 22), GLRLM (*n* = 16); GLSZM (*n* = 16), NGTDM (*n* = 5) and GLDM (*n* = 14). The number of reproducible HRFs varied across kernels and scanner models. Six HRFs were found to be robust to changes in convolution kernels across all scanner models: “Firstorder_10Percentile”, “Firstorder_Energy”, “Firstorder_Mean”, “Firstorder_Median”, “Firstorder_RootMeanSquared” and “Firstorder_TotalEnergy”.

**Figure 2:**
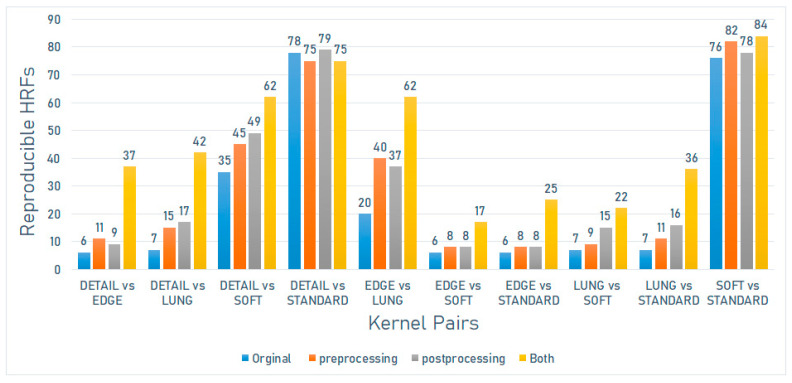
The number of reproducible HRFs across different kernels on the Discovery STE scanner model.

On the Discovery STE scanner model (GE Medical Systems), the number of reproducible HRFs varied between 6 (6.59%) and 78 (85.71%). The greatest number of reproducible HRFs was observed across scans acquired with Detailed and Standard kernels (Figure 2).

**Figure 3:**
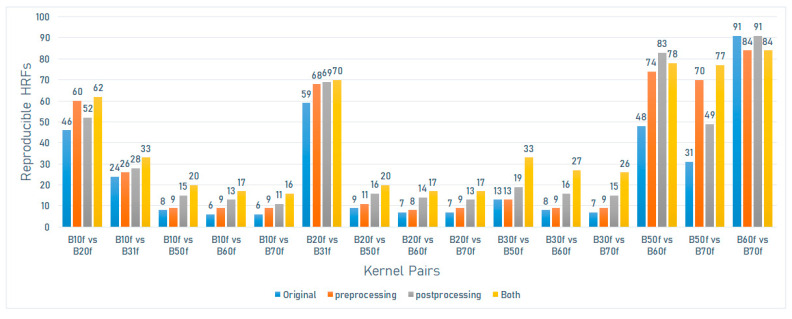
The number of reproducible HRFs across different kernels on the Sensation 40 scanner model.

On the Sensation 40 scanner model (Siemens), the number of reproducible HRFs varied between 6 (6.59%) and 91 (100%). The greatest number of reproducible HRFs was observed across scans acquired with B60f and B70f kernels (Figure 3).

**Figure 4:**
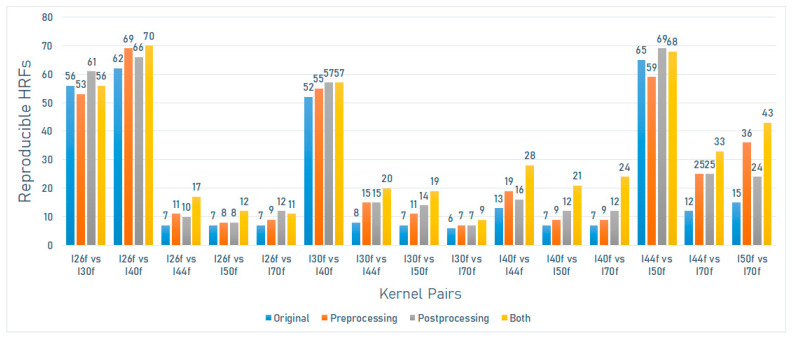
The number of reproducible HRFs across different kernels on the SOMATOM Definition scanner model.

On the SOMATOM definition scanner model (Siemens), the number of reproducible HRFs varied between 6 (6.59%) and 65 (71.4%). The greatest number of reproducible HRFs was observed across scans acquired with I44f and I50f kernels (Figure 4).

**Figure 5:**
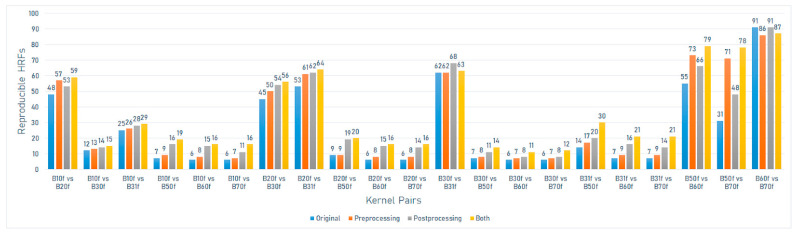
The number of reproducible HRFs across different kernels on the Sensation 64 scanner model.

On the Sensation 64 scanner model (Siemens), the number of reproducible HRFs varied between 6 (6.59%) and 91 (100%). The greatest number of reproducible HRFs was observed across scans acquired with B60f and B70f kernels (Figure 5).

**Figure 6:**
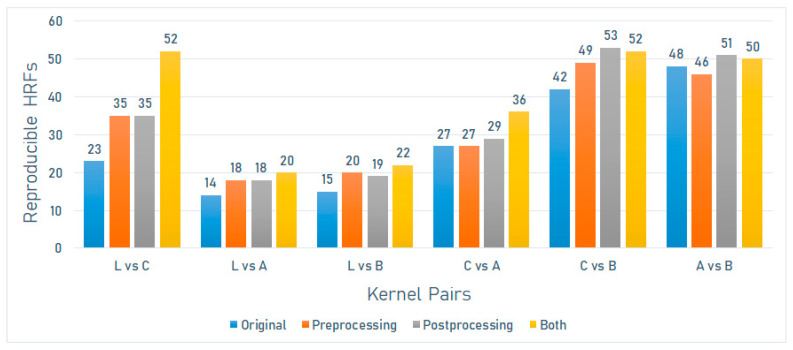
The number of reproducible HRFs across different kernels on the Brilliance 64 scanner model.

On the Brilliance 64 scanner model (Philips), the number of reproducible HRFs varied between 14 (15.4%) and 48 (52.7%). The greatest number of reproducible HRFs was observed across scans acquired with A and B kernels (Figure 6).

### 3.2. The Effects of Pre- and Post-Processing

#### 3.2.1. Reconstruction Kernel Normalization (RKN)

The number of HRFs that became reproducible following the application of the described method varied with the variations in kernels being harmonized and the scanner model used. In most of the investigated scenarios (58 out of 67; 86.6%), the use of this method has resulted in an increment in the number of reproducible HRFs. However, only 19 scenarios (28.4%) showed statistically significant increments. In a number of scenarios (6 out of the analyzed 67 scenarios (9%)), there was a net loss in the number of reproducible HRFs compared to the original, 2 (3%) of which were statistically significant (Figure 2, Figure 3, Figure 4, Figure 5 and Figure 6). In three (4.5%) scenarios, there was no difference between the number of reproducible HRFs extracted from the original and the normalized images.

On the Discovery STE scanner model (GE Medical Systems), the number of reproducible HRFs extracted from the scans after image pre-processing varied between 8 (8.8%) and 82 (90.1%). The greatest increment in the number of reproducible HRFs compared to the original images was observed across scans acquired with Edge and Lung kernels (Figure 2).

On the Sensation 40 scanner model (Siemens), the number of reproducible HRFs extracted from the scans after image pre-processing varied between 8 (8.8%) and 84 (92.3%). In this scenario, the highest number of reproducible HRFs decreased compared to those extracted from the original images for the scans acquired with B60f and B70f. The greatest increment in the number of reproducible HRFs compared to the original images was observed across scans acquired with B50f and B70f kernels (Figure 3).

On the SOMATOM definition scanner model (Siemens), the number of reproducible HRFs extracted from the scans after image pre-processing varied between 7 (7.7%) and 69 (75.8%). The greatest increment in the number of reproducible HRFs compared to the original images was observed across scans acquired with I50f and I70f kernels (Figure 4).

On the Sensation 64 scanner model (Siemens), the number of reproducible HRFs extracted from the scans after image pre-processing varied between 7 (7.7%) and 86 (94.5%). In this scenario, the highest number of reproducible HRFs decreased compared to those extracted from the original images (B60f vs. B70f) (Figure 5).

On the Brilliance 64 scanner model (Philips), the number of reproducible HRFs extracted from the scans after image pre-processing varied between 18 (19.8%) and 49 (53.8%). The greatest increment in the number of reproducible HRFs compared to the original images was observed across scans acquired with L and C kernels (Figure 6).

#### 3.2.2. ComBat Harmonization

In 65 out of the 67 investigated scenarios (97%), there was a net increase in the number of reproducible HRFs compared to the original, with 36 (53.7%) scenarios witnessing significant statistical increments. In two scenarios, the same number of reproducible HRFs was found before and after ComBat harmonization. In 46 (68.7%) scenarios, ComBat harmonization outperformed the RKN method, 17 (25.4%) of which were statistically significant. In 13 (19.4%) scenarios, the RKN method outperformed ComBat harmonization, 5 (7.5%) of which were statistically significant increments.

On the Discovery STE scanner model (GE Medical Systems), the number of reproducible HRFs extracted from the scans after ComBat harmonization varied between 9 (9.9%) and 79 (86.8%). The greatest increment in the number of reproducible HRFs compared to the original images was observed across scans acquired with Edge and Lung kernels (Figure 2).

On the Sensation 40 scanner model (Siemens), the number of reproducible HRFs extracted from the scans after ComBat harmonization varied between 11 (12.1%) and 69 (75.8%). The greatest increment in the number of reproducible HRFs compared to the original images was observed across scans acquired with B50f and B60f kernels (Figure 3).

On the SOMATOM definition scanner model (Siemens), the number of reproducible HRFs extracted from the scans after ComBat harmonization pre-processing varied between 7 (7.7%) and 69 (75.8%). The greatest increment in the number of reproducible HRFs compared to the original images was observed across scans acquired with I44f and I70f kernels (Figure 4).

On the Sensation 64 scanner model (Siemens), the number of reproducible HRFs extracted from the scans after ComBat harmonization varied between 8 (8.8%) and 91 (100%). The greatest increment in the number of reproducible HRFs compared to the original images was observed across scans acquired with B50f and B70f kernels (Figure 5).

On the Brilliance 64 scanner model (Philips), the number of reproducible HRFs extracted from the scans after ComBat harmonization varied between 18 (19.8%) and 53 (58.8%). The greatest increment in the number of reproducible HRFs compared to the original images was observed across scans acquired with L and C kernels (Figure 6).

#### 3.2.3. The Combination of Pre- and Post-Processing

In 63 (95.5%) out of the 67 investigated scenarios, there was a net increase in the number of reproducible HRFs compared to the original, 53 (79.1%) of which were statistically significant. Three (4.5%) showed a lower number of reproducible HRFs, with one (1.5%) scenario showing significantly fewer (*p* < 0.05). The same number of reproducible HRFs was observed in one (1.5%) scenario. In 66 (98.5%) scenarios, the combination of methods outperformed the RKN method, with 42 (62.7%) being significantly higher. The same number of reproducible HRFs was observed in one (1.5%) scenario. With regards to ComBat harmonization, the combination of methods resulted in a higher number of reproducible HRFs in 56 (83.6%) scenarios, 27 (40.3%) of which were statistically significant. A higher number of reproducible HRFs was obtained using only ComBat harmonization in 10 (14.9%) scenarios, only one (1.5%) of which was statistically significant. The same number of reproducible HRFs was observed in one (1.5%) scenario.

On the Discovery STE scanner model (GE Medical Systems), the number of reproducible HRFs extracted from the normalized scans after ComBat harmonization varied between 17 (18.7%) and 84 (92.3%). The greatest increment in the number of reproducible HRFs compared to the original images was observed across scans acquired with Edge and Lung kernels (Figure 2).

On the Sensation 40 scanner model (Siemens), the number of reproducible HRFs extracted from the normalized scans after ComBat harmonization varied between 16 (17.6%) and 84 (92.3%). The greatest increment in the number of reproducible HRFs compared to the original images was observed across scans acquired with B50f and B70f kernels (Figure 3).

On the SOMATOM definition scanner model (Siemens), the number of reproducible HRFs extracted from the normalized scans after ComBat harmonization pre-processing varied between 9 (9.9%) and 70 (77%). The greatest increment in the number of reproducible HRFs compared to the original images was observed across scans acquired with I50f and I70f kernels (Figure 4).

On the Sensation 64 scanner model (Siemens), the number of reproducible HRFs extracted from the normalized scans after ComBat harmonization varied between 11 (12.1%) and 87 (95.7%). The greatest increment in the number of reproducible HRFs compared to the original images was observed across scans acquired with B50f and B70f kernels (Figure 5).

On the Brilliance 64 scanner model (Philips), the number of reproducible HRFs extracted from the normalized scans after ComBat harmonization varied between 20 (22%) and 52 (57.2%). The greatest increment in the number of reproducible HRFs compared to the original images was observed across scans acquired with L and C kernels (Figure 6).

## 4. Discussion

In this study, we analyzed the effects of difference in convolution kernels on five different scanner models, when all other CT acquisition and reconstruction parameters were fixed on a phantom dataset. We further investigated the ability of an image pre-processing (iterative normalization by frequency decomposition) method, and an HRF post-processing harmonization (using ComBat harmonization) method. Our results showed significant differences in the number of reproducible HRFs across the investigated scenarios. Scans reconstructed with similar convolution kernels showed a higher number of reproducible HRFs compared to scans reconstructed with significantly different convolution kernels. Similarly, the performance of both harmonization methods investigated varied with the differences in convolution kernels of the scans being harmonized.

Siemens scanner models (Sensation 40 and 64) have shown the reproducibility of all HRFs across the scans acquired with the higher end of convolution kernels (B60 and B70). Convolution kernels at the opposite end of the spectrum (for example, B10 and B70 on Siemens scanners) have shown the lowest number of reproducible HRFs. As such, our results are in line with previous studies that reported that the reproducibility of HRFs can be significantly affected by variations in convolution kernels [38,58,59,60].

The use of the RKN method on our dataset has resulted in a range of effects on the number of reproducible features, from negative to neutral to positive, depending on the scans being compared. We have observed a significant increase in the number of reproducible HRFs in most scenarios and a decrease in the number of reproducible HRFs in some other scenarios. This could be justified by the possibility that the analyzed data in this study included a wider range of convolution kernels than those used to develop the method.

The application of ComBat harmonization resulted in a higher number of reproducible HRFs compared to those before harmonization in almost all of the investigated scenarios, which is in line with previous reports [43,44,61]. Moreover, on average, ComBat harmonization outperformed the image pre-processing method. The performance of ComBat further depended on the differences in the convolution kernels of the scans being harmonized. In general, the number of reproducible HRFs after ComBat harmonization followed a similar pattern to that of the number of reproducible HRFs before post-processing. These findings are in line with previous studies that investigated the applicability of ComBat harmonization in radiomics analyses [31,34]. The results add to the evidence on the need for reproducibility analyses in radiomics studies, including scans acquired differently, as well as the need for radiomics-specific harmonization methods.

The combination of RKN and ComBat harmonization methods resulted in a higher number of reproducible HRFs across the majority of the investigated scenarios. This indicates that each method could be addressing the reproducibility of HRFs in different manners, with their having been shown to be complementary to each other in many of the investigated scenarios. Nevertheless, the combination resulted in a lower number of reproducible HRFs in an appreciated percentage of scenarios compared to ComBat harmonization only. This suggests the need for reproducibility analysis before applying harmonization methods in radiomics analyses.

We identified six HRFs that were robust with respect to variations in convolution kernels across all the investigated scenarios. These HRFs were first-order statistics, and their robustness could be justified by the standardization of HUs across scanners. However, the majority of texture HRFs were sensitive to the majority of variations in convolution kernels. Clear to the eye, the standardization of image acquisition and reconstruction parameters would be the cornerstone for the translation of radiomic signatures to clinical practice. The findings of this study, and previous experiments, have shown that the reproducibility of HRFs significantly depends on imaging acquisition and reconstruction parameters. Therefore, reproducibility analysis is needed for a proper understanding of their performance or generalizability [19]. Another potential solution would be the development of radiomic signatures specific to a set of imaging acquisition and reconstruction parameters. However, this solution limits the generalizability of radiomic signatures.

While we tried to analyze all the kernels used in clinical practice, we were limited by the available data. However, the results have shown a similar pattern across different scanner models. Future studies that include a wider spectrum of convolution kernels are recommended. Furthermore, we limited our analyses to the original HRFs as they are commonly standardized across radiomics platforms. Detailed full HRF reproducibility analysis could be beneficial for specific tasks. Furthermore, the analysis was performed on a phantom dataset that was designed to mimic human tissues. However, it only gives an idea about the reproducibility of HRFs in the given scenarios, and similar analysis is needed for patient datasets to gain a full understanding. The potential of other harmonization methods, for example, dynamic range limitation [62], could also be explored in future studies. Additionally, the sensitivity of HRFs to variations in segmentations could not be assessed in this study, due to the use of automated segmentations.

## 5. Conclusions

The reproducibility of the majority of HRFs depended on the variations in reconstruction kernels in the data being analyzed. Six HRFs were found to be reproducible across all investigated scenarios. Radiomics analysis of scans acquired with different reconstruction kernels is not recommended in the absence of reproducibility analysis. We recommend the systematic use of RKN and ComBat harmonization in future radiomics studies, including images acquired similarly except for the reconstruction kernel. Nevertheless, their application should follow a reproducibility analysis to identify the set of reproducible HRFs after harmonization. HRF-specific harmonization methods remain necessities in the field of radiomics.

## Figures and Tables

**Table 1:** Acquisition and reconstruction parameters for the imaging dataset.

Manufacturer	Scanner Model	Number of Scans	X-ray Tube Current (kV)	Convolution Kernels	Slice Thickness (mm)	Pixel Spacing (mm^2^)
GE	Discovery STE	5	120	Standard, Detail, Edge, Soft, Lung	1.25	0.49 × 0.49
Philips	Brilliance 64	4	120	A, B, C, L	1.50	0.49 × 0.49
Siemens	Sensation 40	6	120	B10f, B20f, B31f, B50f, B60f, B70f	1.50	0.49 × 0.49
	Sensation 64	7	120	B10f, B20f, B30f, B31f, B50f, B60f, B70f	1.50	0.49 × 0.49
	SOMATOM Definition AS	6	120	I26f, I30f, I40f, I44f, I50f, I70f	1.50	0.49 × 0.49

## Data Availability

The data presented in this study are openly available at TCIA.org: http://doi.org/10.7937/K9/TCIA.2017.zuzrml5b (accessed on 7 January 2021).
